# Methylmercury Induces Apoptosis in Mouse C17.2 Neural Stem Cells through the Induction of OSGIN1 Expression by NRF2

**DOI:** 10.3390/ijms25073886

**Published:** 2024-03-30

**Authors:** Naoya Yamashita, Marino Uchiyama, Ryota Yamagata, Gi-Wook Hwang

**Affiliations:** Laboratory of Environmental and Health Sciences, Faculty of Pharmaceutical Sciences, Tohoku Medical and Pharmaceutical University, 4-4-1 Komatsushima, Aoba-ku, Sendai 981-8558, Miyagi, Japan; yamashita@tohoku-mpu.ac.jp (N.Y.); yamagata@tohoku-mpu.ac.jp (R.Y.)

**Keywords:** methylmercury, OSGIN1, apoptosis, toxicity

## Abstract

Methylmercury is a known environmental pollutant that exhibits severe neurotoxic effects. However, the mechanism by which methylmercury causes neurotoxicity remains unclear. To date, we have found that oxidative stress-induced growth inhibitor 1 (OSGIN1), which is induced by oxidative stress and DNA damage, is also induced by methylmercury. Therefore, in this study, we investigated the relationship between methylmercury toxicity and the induction of OSGIN1 expression using C17.2 cells, which are mouse brain neural stem cells. Methylmercury increased both OSGIN1 mRNA and protein levels in a time- and concentration-dependent manner. Moreover, these increases were almost entirely canceled out by pretreatment with actinomycin D, a transcription inhibitor. Furthermore, similar results were obtained from cells in which expression of the transcription factor nuclear factor erythroid 2-related factor 2 (NRF2) was suppressed, indicating that methylmercury induces OSGIN1 expression via NRF2. Methylmercury causes neuronal cell death by inducing apoptosis. Therefore, we next investigated the role of OSGIN1 in methylmercury-induced neuronal cell death using the activation of caspase-3, which is involved in apoptosis induction, as an indicator. As a result, the increase in cleaved caspase-3 (activated form) induced by methylmercury exposure was decreased by suppressing OSGIN1, and the overexpression of OSGIN1 further promoted the increase in cleaved caspase-3 caused by methylmercury. These results suggest, for the first time, that OSGIN1 is a novel factor involved in methylmercury toxicity, and methylmercury induces apoptosis in C17.2 cells through the induction of OSGIN1 expression by NRF2.

## 1. Introduction

Methylmercury is a type of organic alkylmercury compound known to exhibit serious neurotoxic effects as an environmental pollutant [1,2,3,4]. The global circulation of mercury is well known; mercury vapor released into the environment through volcanic activity becomes inorganic mercury, which accumulates in soil and aquatic environments through rainfall [5]. The chemical conversion of inorganic mercury to methylmercury is the first step in the accumulation process in the hydrosphere, and the reaction involves either non-enzymatic methylation or biological methylation by microorganisms. Therefore, methylmercury ubiquitously exists in the environment, regardless of environmental pollution. In recent years, large amounts of mercury have been released into the environment due to gold mining and other activities around the Amazon in Brazil, and high concentrations of methylmercury have accumulated in the bodies of populations living in these areas, raising concerns about the health effects [6,7,8]. In addition, it has been suggested that children born from pregnancies during which relatively large amounts of methylmercury were ingested through seafood may develop intellectual developmental disorders [9,10,11]. Thus, the effects of methylmercury exposure on fetal neurodevelopment are considered to be a problem around the world. However, the detailed mechanisms involved in toxicity of methylmercury remain unclear.

We comprehensively searched for intracellular factors involved in methylmercury toxicity using various search tools, and identified tumor necrosis factor α [12], homeobox B13 [13], and other factors involved in enhancing methylmercury toxicity. In contrast, we also reported that chemokine ligand 3 [14], ornithine decarboxylase 1 (ODC1) [15,16], transcription factor 3 [17], and other factors are involved in reducing methylmercury toxicity. However, because methylmercury is expected to exert its own toxicity by affecting various intracellular factors, the mechanisms involved in methylmercury toxicity have not yet been completely elucidated. To simulate such a situation, we exposed C17.2 cells derived from mouse brain neural stem cells to methylmercury and analyzed gene expression changes using RNA sequencing (RNA-seq). As a result, we found that methylmercury induces the expression of oxidative stress-induced growth inhibitor 1 (OSGIN1; also known as OKL38), a gene whose expression is induced by oxidative stress and DNA damage (unpublished data). Other research groups have also previously found that methylmercury induces OSGIN1 expression [18,19,20], but the relationship between methylmercury toxicity and OSGIN1 has not been investigated.

It is well known that apoptosis is involved in methylmercury-induced neurotoxicity [3,21,22]. We and other researchers have shown that apoptosis via caspase-3 activation is involved in methylmercury-induced neuronal cell death [14,15,17,23,24,25]. We have also revealed that methylmercury induces apoptosis in C17.2 cells by activating the cytochrome c/caspase-9/caspase-3 pathway through mitochondrial damage and reactive oxygen species (ROS) production [15]. In this study, we aimed to clarify the role of OSGIN1 in methylmercury toxicity and investigated the mechanism by which methylmercury induces OSGIN1 expression and the involvement of OSGIN1 in methylmercury-induced apoptosis as an indicator of caspase-3 activation.

## 2. Results

### 2.1. Methylmercury Induces OSGIN1 Expression in C17.2 Cells

**Figure 1:**
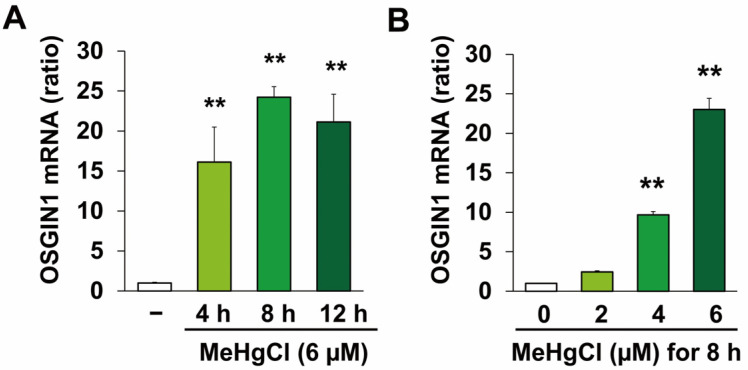
Effects of methylmercury on OSGIN1 mRNA expression. C17.2 cells (4 × 10^4^ cells/well) were seeded onto 24-well plates for 24 h. (**A**) Cells were exposed for the indicated period to methylmercury chloride (MeHgCl) (6 µM) or (**B**) exposed to the indicated concentration of MeHgCl for 8 h. mRNA levels of OSGIN1 and GAPDH were measured, and the relative values normalized to GAPDH are shown (*n* = 3). The data are represented as mean ± SD. ** *p* < 0.01.

**Figure 2:**
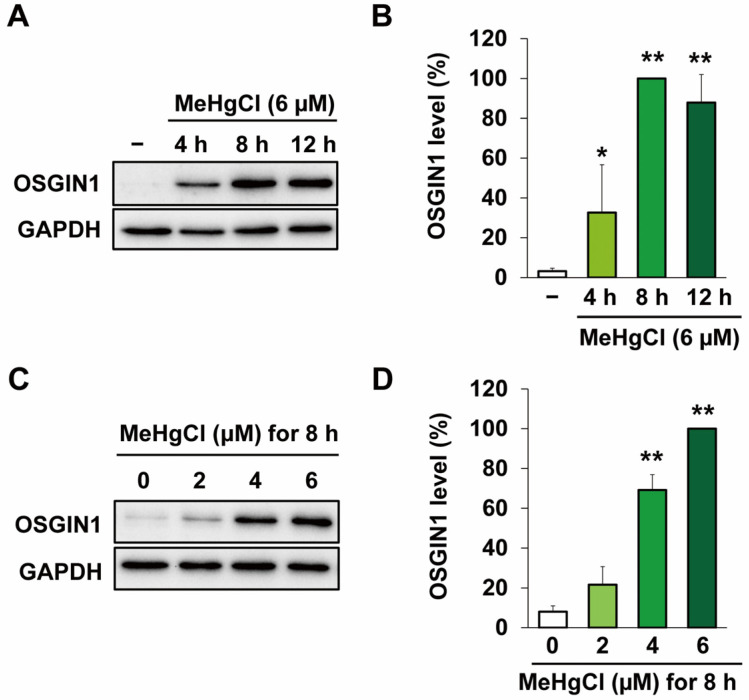
Effects of methylmercury on OSGIN1 protein expression. C17.2 cells (4 × 10^4^ cells/well) were seeded onto 24-well plates for 24 h. (**A**,**B**) Cells were exposed for the indicated period to methylmercury chloride (MeHgCl) (6 µM) or (**C**,**D**) exposed to the indicated concentration of MeHgCl for 8 h. OSGIN1 protein levels were examined by Western blotting (**A**,**C**) and quantification of the band intensity of OSGIN1 [the band intensity of cells exposed to MeHgCl for 8 h (**B**), 6 μM, (**D**) was considered as 100%, normalized to each GAPDH level] shown in (**B**,**D**). The data are represented as mean ± SD. ** *p* < 0.01, * *p* < 0.05.

We first examined OSGIN1 gene expression under conditions of exposure to methylmercury at a concentration that is minimally cytotoxic to C17.2 cells. Further, the concentrations and times of methylmercury exposure were based on the results of previous studies [13,15,16] and preliminary experiments to evaluate methylmercury-induced apoptosis (Figure S1). As a result, exposure to methylmercury at a final concentration of 6 μM increased OSGIN1 mRNA levels, with the maximum peak in particular being observed after 8 h of exposure (Figure 1A). Methylmercury also increased OSGIN1 mRNA levels in a concentration-dependent manner (Figure 1B). Next, we examined the effects of methylmercury on OSGIN1 protein expression. As a result, OSGIN1 protein levels were increased following methylmercury exposure in a time- (Figure 2A,B) and concentration-dependent manner (Figure 2C,D). The above results indicate that methylmercury may increase the expression of OSGIN1 mRNA and protein prior to cell death.

### 2.2. Methylmercury Induces OSGIN1 Expression by Promoting Its Transcription

**Figure 3:**
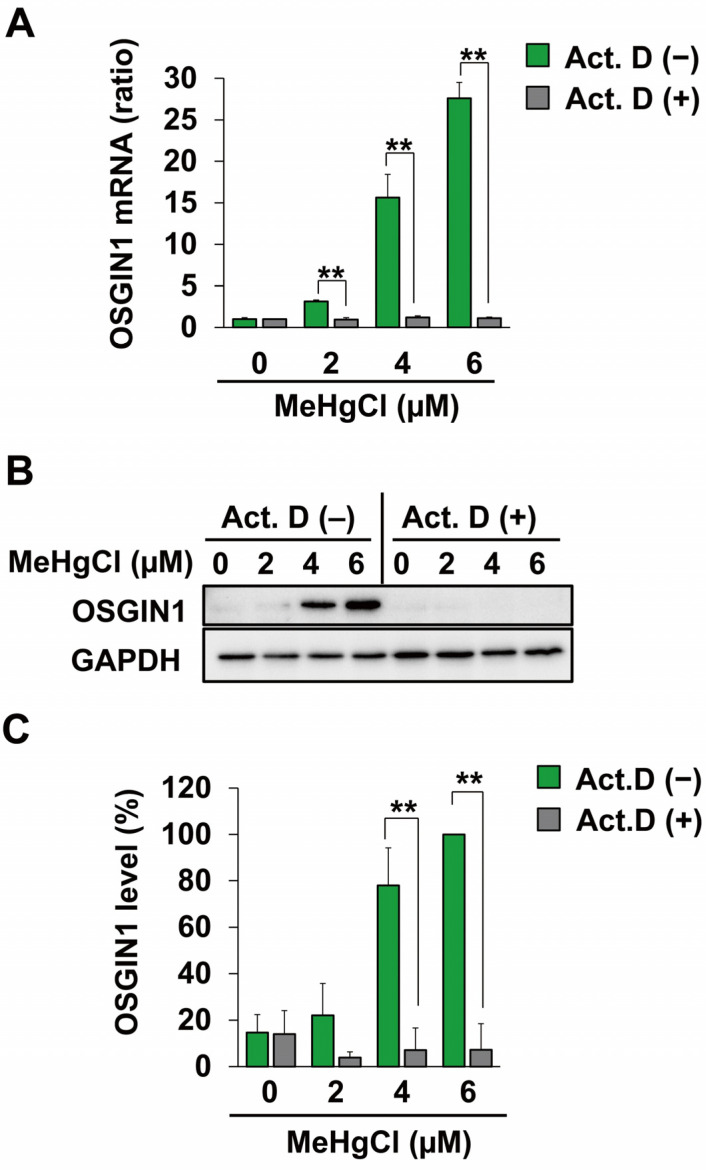
Effects of a transcription inhibitor on induction of OSGIN1 expression by methylmercury. C17.2 cells (4 × 10^4^ cells/well) were seeded onto 24-well plates for 23 h. C17.2 cells were pretreated with 1 µM of actinomycin D (Act. D) for 1 h and exposed to the indicated concentration of methylmercury chloride (MeHgCl) for 8 h. (**A**) mRNA levels of OSGIN1 and GAPDH were measured, and relative values normalized to GAPDH are shown. OSGIN1 protein levels were examined by Western blotting (**B**). Quantification of the band intensity of OSGIN1 [the band intensity of control cells exposed to MeHgCl (6 μM) was considered as 100%, normalized to each GAPDH level] shown in (**C**). The data are represented as mean ± SD. ** *p* < 0.01.

Because an increase in OSGIN1 mRNA levels caused by methylmercury was observed in Figure 1, we examined the effects of the transcriptional inhibitor actinomycin D (Act.D) on this increase. As a result, pretreatment with Act.D resulted in almost no increase in OSGIN1 mRNA levels caused by methylmercury (Figure 3A). Furthermore, under the same conditions, the increase in OSGIN1 protein levels caused by methylmercury was also successfully canceled out (Figure 3B,C). These results strongly suggest that methylmercury induces the expression of OSGIN1 mRNA and protein by promoting their transcription.

### 2.3. Methylmercury Induces OSGIN1 Expression in C17.2 Cells in an NRF2-Dependent Manner

**Figure 4:**
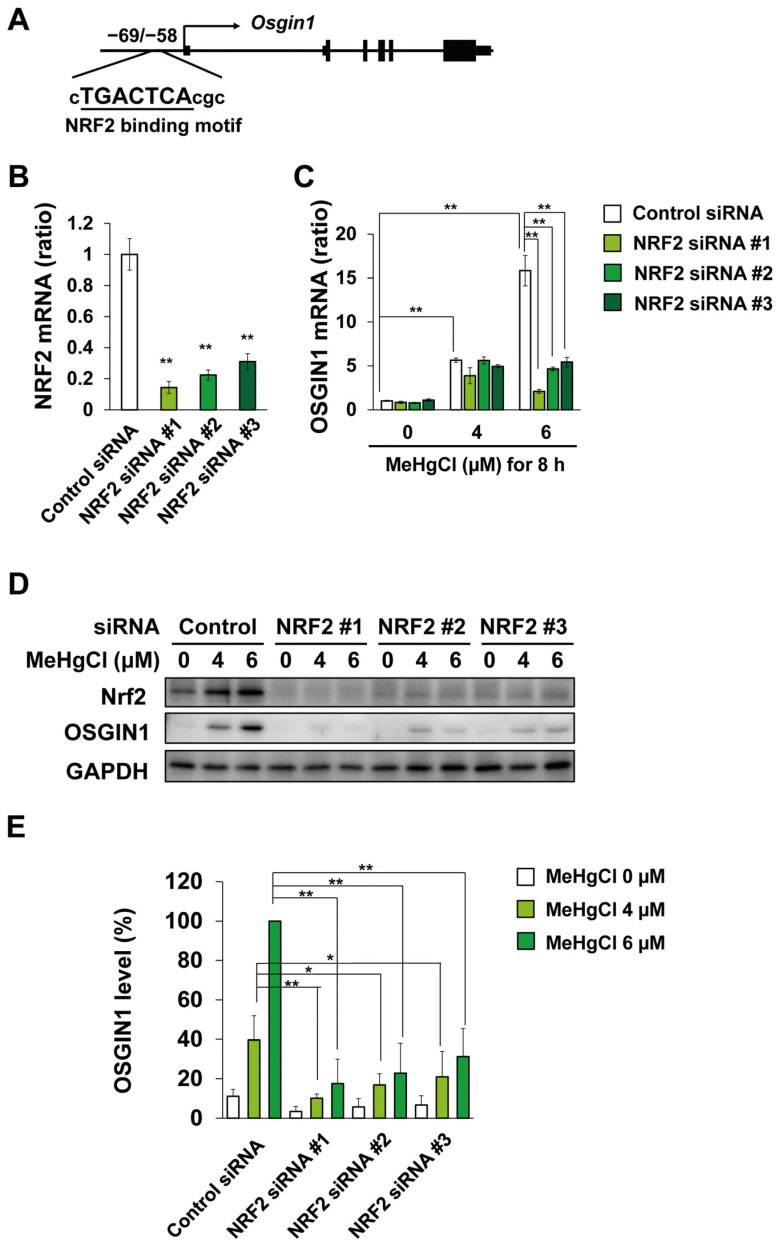
Effects of NRF2 knockdown on the induction of OSGIN1 expression by methylmercury. (**A**) Schematic representation of OSGIN1 gene locus. Putative NRF2 binding sequences are underlined. C17.2 cells (1.5 × 10^4^ cells/well) were seeded onto 24-well plates for 24 h. C17.2 cells were transfected with control siRNA or NRF2 siRNA for 24 h. Cells were then exposed to the indicated concentration of methylmercury chloride (MeHgCl) for 8 h. mRNA levels of NRF2 (**B**), OSGIN1 (**C**), and GAPDH were measured, and relative values normalized to GAPDH are shown. OSGIN1 and NRF2 protein levels were examined by Western blotting (**D**). Quantification of the band intensity of OSGIN1 [the band intensity of control cells exposed to MeHgCl (6 μM) was considered as 100%, normalized to each GAPDH level] shown in (**E**). The data are represented as mean ± SD. ** *p* < 0.01, * *p* < 0.05.

Until now, nuclear factor erythroid 2-related factor 2 (NRF2) has been reported as a transcription factor involved in the induction of OSGIN1 expression [26,27], and the partial binding sequence of NRF2 is located −69 to −58 bp from the transcription start site of the OSGIN1 gene (Figure 4A). It has also been reported that methylmercury activates NRF2 in various cell types and mice [28,29,30,31]. Therefore, to clarify the involvement of NRF2 in OSGIN1 induction by methylmercury, we transfected C17.2 cells with three types of siRNA against NRF2 mRNA and examined the effects of methylmercury exposure on OSGIN1 expression. As a result, the NRF2 mRNA levels were decreased in C17.2 cells transfected with either siRNA, and the increase in OSGIN1 mRNA levels caused by methylmercury was significantly reduced in these cells (Figure 4B,C). Furthermore, the increase in OSGIN1 protein levels caused by methylmercury was virtually no longer observed in all cells in which NRF2 expression was suppressed (Figure 4D,E). These findings strongly suggest that methylmercury induces the expression of OSGIN1 via NRF2.

### 2.4. OSGIN1 Is Involved in Apoptosis Caused by Methylmercury

**Figure 5:**
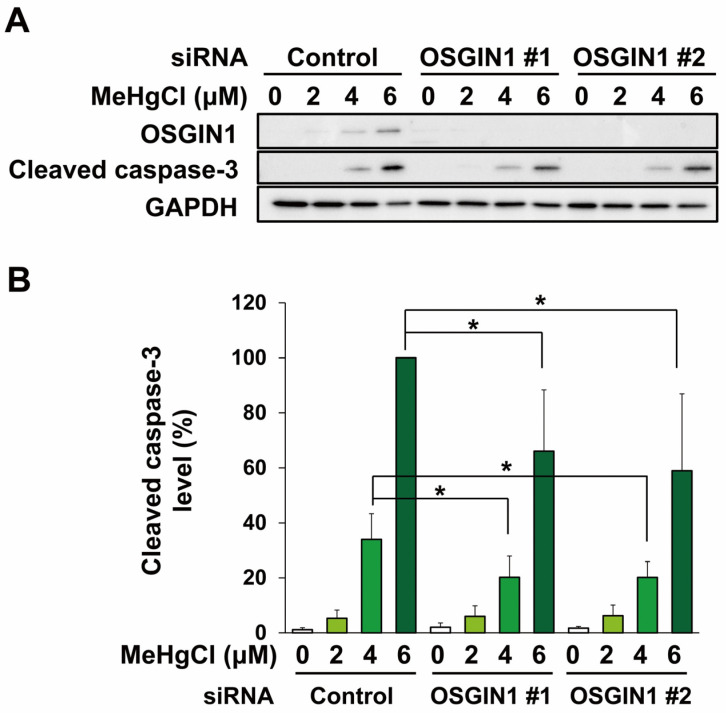
Effects of knockdown of OSGIN1 on methylmercury-induced apoptosis. C17.2 cells (1.5 × 10^4^ cells/well) were seeded onto 24-well plates for 24 h. C17.2 cells were transfected with control siRNA or OSGIN1 siRNA for 24 h. Cells were then exposed to the indicated concentration of methylmercury chloride (MeHgCl) for 24 h. OSGIN1 and cleaved caspase-3 protein levels were examined by Western blotting (**A**). Quantification of the band intensity of cleaved caspase-3 [the band intensity of control cells exposed to MeHgCl (6 μM) was considered as 100%, normalized to each GAPDH level] shown in (**B**). The data are represented as mean ± SD. * *p* < 0.05.

**Figure 6:**
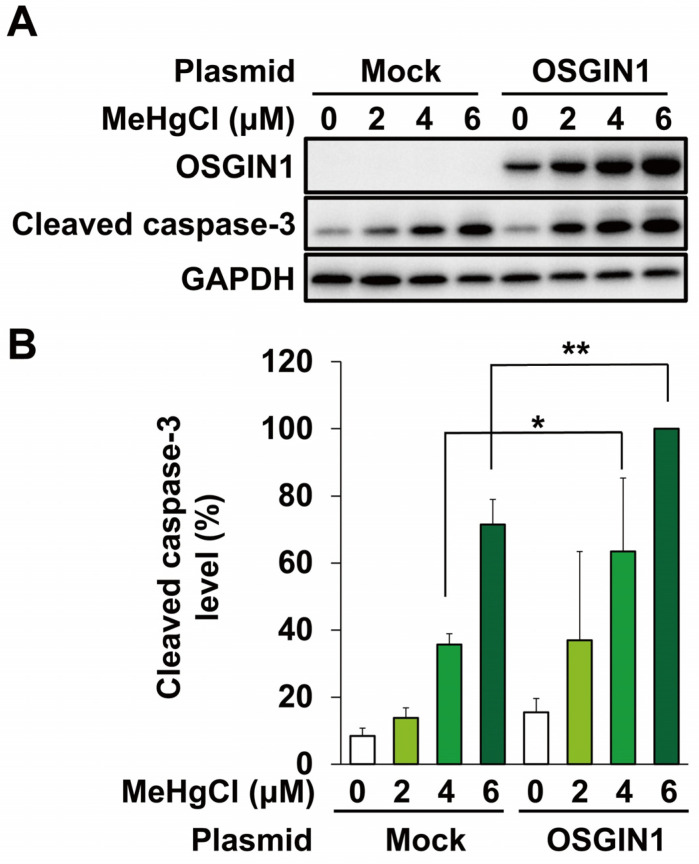
Effects of overexpression of OSGIN1 on methylmercury-induced apoptosis. C17.2 cells (2 × 10^4^ cells/well) were seeded onto 24-well plates for 24 h. C17.2 cells were transfected with empty vector (Mock) or OSGIN1-expressing plasmid for 24 h. Cells were then exposed to the indicated concentration of methylmercury chloride (MeHgCl) for 24 h. (**A**) OSGIN1 and cleaved caspase-3 protein levels were examined by Western blotting. Quantification of the band intensity of cleaved caspase-3 [the band intensity of control cells exposed to MeHgCl (6 μM) was considered as 100%, normalized to each GAPDH level] shown in (**B**). The data are represented as mean ± SD. ** *p* < 0.01, * *p* < 0.05.

Methylmercury is known to cause neuronal cell death by inducing apoptosis, and it has been reported that methylmercury also induces apoptosis in C17.2 cells [15,17]. Therefore, we used two types of siRNA against OSGIN1 mRNA to examine the effects of suppressing OSGIN1 expression on methylmercury-induced apoptosis in C17.2 cells. In this study, we evaluated the levels of cleaved caspase-3, which is an activated form of caspase-3 involved in apoptosis induction. As a result, cleaved caspase-3 appeared following exposure to 4 µM methylmercury, the conditions under which OSGIN1 expression induction was observed, and further increased when exposed to 6 µM (Figure 5A,B). In addition, the increase in cleaved caspase-3 levels caused by methylmercury was reduced in both OSGIN1-knockdown cell preparations (Figure 5A,B). Furthermore, to clarify the involvement of increased OSGIN1 in apoptosis, C17.2 cells were transfected with an OSGIN1 expression plasmid. As a result, cleaved caspase-3 levels tended to increase due to the overexpression of OSGIN1, and a significant increase in cleaved caspase-3 levels due to methylmercury was observed in these cells compared with control cells (Figure 6A,B). The above results suggest that methylmercury promotes apoptosis in C17.2 cells through the induction of OSGIN1 expression.

## 3. Discussion

In this study, we used C17.2 cells, which are mouse brain neural stem cells, and found that methylmercury induces apoptosis by promoting OSGIN1 transcription through the activation of the transcription factor NRF2.

It has been reported that doxorubicin, an anticancer drug, activates the tumor suppressor gene p53 and promotes the transcription of OSGIN1 [32], and that oxidative stress caused by exposure to cigarette smoke and particulate matter 2.5 (PM_2.5_) increases OSGIN1 expression [33,34]. It is also known that docosahexaenoic acid (DHA) activates NRF2 through ROS production and induces OSGIN1 expression in human breast cancer cell lines (MCF-7) [35]. In Figure 4, the induction of OSGIN1 expression by methylmercury was almost entirely canceled out by the suppression of NRF2 expression, suggesting that NRF2 is a major transcription factor involved in promoting OSGIN1 transcription in C17.2 cells. Methylmercury is known to directly bind to the 151st cysteine residue of Kelch-like ECH-associated protein 1 (KEAP1) [29], known as a negative regulator of NRF2, and activate it by suppressing the degradation of NRF2 due to KEAP1. Furthermore, methylmercury is known to promote ROS production [15,21,36,37], and ROS is also involved in NRF2 activation by suppressing KEAP1 [38]. Therefore, methylmercury is expected to be involved in the activation of NRF2 in C17.2 cells through the aforementioned actions.

Overexpression of a human osteosarcoma cell line (U2OS) to OSGIN1 was shown to increase ROS production and promote apoptosis via cytochrome c being released from mitochondria [32]. It has also been reported that DHA induces apoptosis by promoting ROS production in mitochondria through increased OSGIN1 expression [35,39]. We previously reported that methylmercury induces apoptosis in C17.2 cells mainly by causing mitochondrial damage and ROS generation [15]. Therefore, we examined the effects of OSGIN1 knockdown on methylmercury-induced ROS production. As a result of using three types of siRNAs against OSGIN1 mRNA, methylmercury-induced ROS production was attenuated in one type of OSGIN1-knockdown cells, whereas similar results were not obtained in other cells (Figure S2). This suggests that, at least under the present conditions, OSGIN1 may not be involved in methylmercury-induced ROS production. However, caspase-3 activation by methylmercury was partially suppressed in C17.2 cells in which OSGIN1 expression was suppressed (Figure 5). This suggests that, although OSGIN1 is involved in the apoptosis-inducing pathway mediated by mitochondrial damage, there may also be an OSGIN1-independent pathway in C17.2 cells. On the other hand, it has been shown that the induction of OSGIN1 expression through activation of NRF2 by monomethyl fumarate attenuates hydrogen peroxide-induced cell death in primary human spinal cord astrocytes [26]. However, under these conditions, the expression of an OSGIN1-splicing variant with a molecular weight of 61 kDa, which is larger than the original molecular weight of 52 kDa, is induced and involved in alleviating cell death caused by hydrogen peroxide. Although the mechanisms of the conflicting actions on cell death among OSGIN1 of different molecular weights are still unknown, it is thought that methylmercury causes apoptosis by inducing the expression of 52-kDa OSGIN1, at least in C17.2 cells.

Until now, NRF2 has been widely known as a factor involved in reducing the toxicity of various chemicals including methylmercury [28,29,40]. However, the results of this study suggest that it enhances methylmercury toxicity by inducing the expression of OSGIN1. In other words, our results show that, at least in C17.2 cells, both reducing and enhancing factors for methylmercury toxicity may be present among the downstream factors of NRF2. Moreover, because methylmercury simultaneously induces the expression of these factors via NRF2, the extent of caspase-3 activation may not have affected the degree of caspase-3 activation.

We recently found in preliminary experiments that the expression of OSGIN1 protein is increased in neurons in the cerebrum of male mice exposed to methylmercury. Interestingly, the results show that an increased expression of OSGIN1 may occur before methylmercury-induced neuronal damage in a mouse brain. These results suggest that methylmercury may cause neuronal damage in the brain by inducing OSGIN1 expression not only in C17.2 cells but also in mice. In the future, we plan to use mice to clarify the relationship between OSGIN1 and neuronal damage in the brain caused by methylmercury.

In this study, we identified OSGIN1 as a novel factor involved in methylmercury-induced neuronal damage for the first time. However, this study only used C17.2 cells and did not examine whether OSGIN1 is also involved in methylmercury toxicity in other cells, such as primary neurons. Furthermore, the mechanisms involved in methylmercury toxicity due to OSGIN1 are still unknown; thus, these are limitations of our study. In the future, elucidating the detailed mechanisms involved in methylmercury toxicity mediated by OSGIN1 in C17.2 cells and primary neurons is expected to lead to the elucidation of the mechanisms of neuronal damage in the brain caused by methylmercury, as well as to the development of therapeutic agents.

## 4. Materials and Methods

### 4.1. Cell Culture

The v-myc immortalized C17.2 cell lines were derived from cloned mouse cerebellar neural stem cells [41] and were obtained from the European Collection of Cell Cultures (ECACC). The cells were cultured in Dulbecco’s Modified Eagle Medium (Nissui Pharmaceutical, Tokyo, Japan) supplemented with 10% fetal bovine serum (Gibco; Thermo Fisher Scientific, Waltham, MA, USA) and 2 mM L-glutamine (Nacalai Tesque, Kyoto, Japan) in a humidified atmosphere of 5% CO_2_ at 37 °C.

### 4.2. Quantitative PCR (qPCR)

Total RNA was isolated by ISOGEN II (Nippon Gene, Tokyo, Japan) according to the manufacturer’s instructions. cDNA was synthesized from total RNA using a PrimeScript RT reagent kit with oligo dT primer (Takara, Shiga, Japan) according to the manufacturer’s instructions. qPCR was performed using SYBR Premix Ex Taq (Takara) with a LightCycler 96 System (Roche Diagnostics, Mannheim, Germany) with the following primers: mouse OSGIN1, F: 5′-AACTTTGGCATTGTGGAAGG-3′, R: 5′-ACACATTGGGGGTAGGAACA-3′; mouse NRF2, F: 5′-CATAGAGCAGGACATGGAGCAAG-3′, R: 5′-CGGTAGTATCAGCCAGCTGCTTG-3′; and mouse GAPDH, F: 5′-CTGCGTCCTGACACAGACTT-3′, R: 5′-GGTCACCATGGAGCCTTCAA-3′. The reaction was performed at a final volume of 10 µL containing 5 µL SYBR Premix Ex Taq, 0.2 µL of each primer (10 µM), 1.5 µL cDNA (5 ng/µL), and Milli-Q water (Merck Millipore, Billerica, MA, USA). The amplification protocol consisted of 40 cycles of qPCR for denaturation for 30 s at 95 °C, and annealing/extension for 30 s at 60 °C for each mRNA. The level of each mRNA was assessed by the relative standard curve method. The data are presented as values corrected by GAPDH.

### 4.3. Western Blotting

The cells were harvested in a 2% sodium dodecyl sulfate (SDS) buffer. Lysates were then incubated at 95 °C for 5 min. The protein concentration of each lysate was examined using a DC Protein Assay Kit (Bio-Rad, Hercules, CA, USA). Whole cell lysates (approximately 15 µg) were separated using SDS-polyacrylamide gel electrophoresis and separated proteins were then transferred to polyvinylidene fluoride membranes (Immobilon P; EMD Millipore, Burlington, MA, USA). The membrane was blocked for 1 h in 5% skimmed milk (Fujifilm-Wako, Osaka, Japan). Immunoblotting was conducted using anti-OSGIN1 (1:1000 dilution; 15248-1-AP, Proteintech, Chicago, IL, USA), anti-NRF2 (1:1000 dilution; 16396-1-AP, Proteintech), anti-cleaved caspase-3 (1:1000 dilution; 9661, Cell Signaling Technologies, Danvers, MA, USA), and anti-GAPDH (1:5000 dilution; 015-25473, Fujifilm-Wako). Horseradish peroxidase-conjugated anti-rabbit IgG (1:10,000 dilution; 458, Medical & Biological Laboratories Co., Ltd., Nagoya, Japan) was used as the secondary antibody. Primary antibodies and secondary antibodies were diluted with Can Get Signal Immunoreaction Enhancer Solution 1 (Toyobo, Osaka, Japan) and Can Get Signal Immunoreaction Enhancer Solution 2 (Toyobo), respectively. Protein band images were acquired using a ChemiDoc Touch Imaging System (Bio-Rad) and analyzed using Image Lab Software (version 5.2.1, Bio-Rad).

### 4.4. siRNA Transfection

The cells (1.5 × 10^4^ cells/well) were seeded on a 24-well plate and cultured for 24 h. The indicated siRNA was then transfected with Lipofectamine RNAiMAX transfection reagent (Thermo Fisher Scientific) according to the manufacturer’s instructions. The cells were cultured for a further 24 h for OSGIN1 or NRF2 knockdown, then exposed to methylmercury at the indicated concentrations. All siRNAs were purchased from Sigma-Aldrich (St. Louis, MO, USA). Forward sequences of siRNA are as follows: OSGIN1 siRNA #1 (5′-CACUGUGAACCCAACCUCAdTdT-3′), OSGIN1 siRNA #2 (5′-GUCAAAGACUGGAUGCGGAdTdT-3′), NRF2 siRNA#1 (5′-GACUCAAAUCCCACCUUAAdTdT-3′), NRF2 siRNA#2 (5′-GUGAAAUGCAGAAACACUUdTdT-3′), and NRF2 siRNA#3 (5′-GAAACCUCCAUCUACUGAAdTdT-3′). Negative control siRNAs were also obtained from Sigma-Aldrich.

### 4.5. Plasmid Construction and Transfection

The mouse OSGIN1 gene was amplified by KOD-plus neo (Toyobo, Osaka, Japan) from the complementary DNA of C17.2 cells using primers [5′-ATCATGACCTCCTGGAGGCACGACTC-3′ (sense) and 5′-TATCTCGAGTTAAGGTGGCTTCCTGGTCTCCT-3′ (antisense)] and inserted between the EcoRV (New England Biolabs, Beverly, MA, USA) and XhoI (New England Biolabs) sites of a myc-tag-containing pcDNA5/TO vector (Invitrogen, Carlsbad, CA, USA). C17.2 cells were transfected with control vector or OSGIN1-expressing plasmid using PEI Max Reagent (Polysciences, Warrington, PA, USA) for 24 h, then exposed to methylmercury at the indicated concentrations.

### 4.6. Statistical Analysis

Multiple group comparisons were performed using a one-way analysis of variance followed by a post hoc Dunnett’s multiple comparison test. Unpaired Student’s t-tests were performed to compare differences between two groups. Statistical analyses were performed using KaleidaGraph software (v4.1.1; Synergy Software, Eden Prairie, MN, USA), with statistical significance being set at *p* < 0.05.

## Data Availability

Please contact the corresponding author for reasonable data request.

## Supplementary Materials

The following supporting information can be downloaded at: https://www.mdpi.com/article/10.3390/ijms25073886/s1.
