# Supplementary material for: Methylmercury Induces Apoptosis in Mouse C17.2 Neural Stem Cells through the Induction of OSGIN1 Expression by NRF2
Source: Int J Mol Sci. 2024 Mar 30;25(7):3886. doi: 10.3390/ijms25073886 (PMC11011283; doi:10.3390/ijms25073886)
Supplement: Supplementary file 1 [file ijms-25-03886-s001.zip › ijms-2915735-supplementary.pdf]

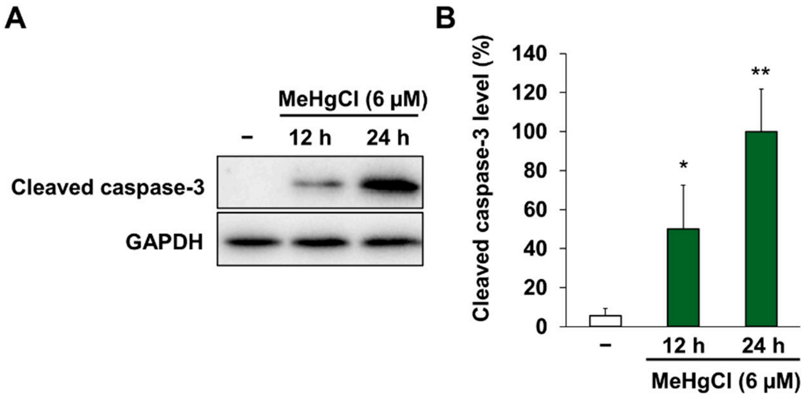

Figure S1. Effects of methylmercury on apoptosis. C17.2 cells (4 × 10^4 cells/well) were seeded onto 24-well plates for 24 h. Cells were then exposed to methylmercury chloride (MeHgCl, 6 μM) for 12 or 24 h. Cleaved caspase-3 and GAPDH protein levels were examined by Western blotting (A). Quantification of the band intensity of cleaved caspase-3 [the band intensity of control cells exposed to MeHgCl (24 h) was considered as 100%, normalized to each GAPDH level] shown in (B). The data are represented as mean ± SD. $^{**}p < 0.01$, $^{*}p < 0.05$.

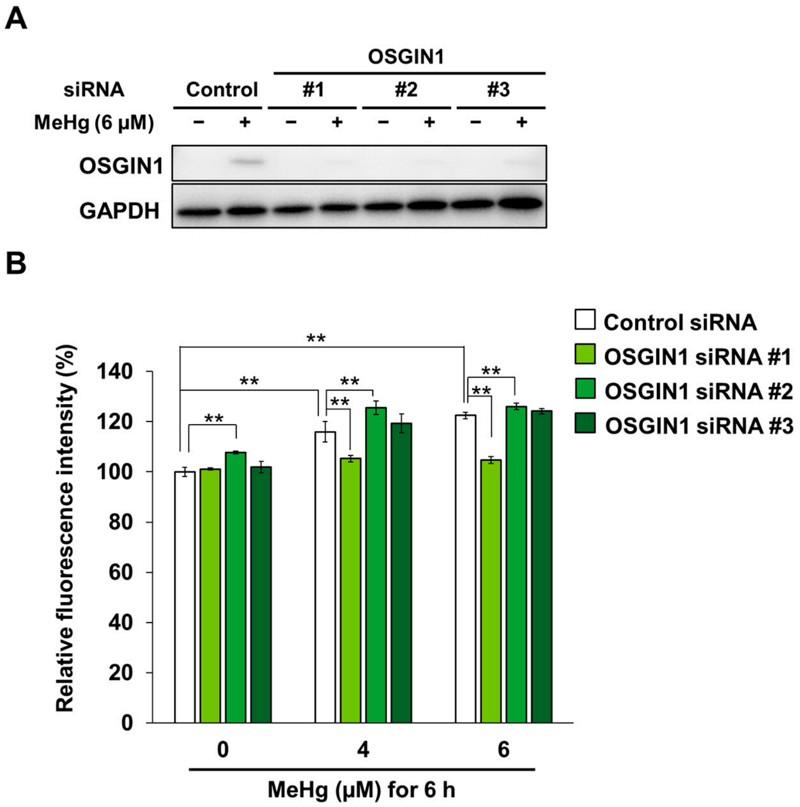

Figure S2. Effects of knockdown of OSGIN1 on methylmercury-induced ROS production. C17.2 cells (1 × 10^4 cells/well) were seeded onto 96-well plates for 24 h. C17.2 cells were transfected with control siRNA or OSGIN1 siRNA for 24 h. Cells were then exposed to the indicated concentration of methylmercury chloride (MeHgCl) for 6 h. OSGIN1 and cleaved caspase-3 protein levels were examined by Western blotting (A). Reactive oxygen species (ROS) were stained with CellROX Green reagent and the fluorescence intensity was analyzed and quantified with the Operetta high-content imaging system. The data are represented as mean ± SD. $^{**}p < 0.01$.
